# Disulfide Bond Engineering for Enhancing the Thermostability of the Maltotetraose-Forming Amylase from *Pseudomonas saccharophila* STB07

**DOI:** 10.3390/foods11091207

**Published:** 2022-04-21

**Authors:** Yinglan Wang, Caiming Li, Xiaofeng Ban, Zhengbiao Gu, Yan Hong, Li Cheng, Zhaofeng Li

**Affiliations:** 1 Key Laboratory of Synergetic and Biological Colloids, Ministry of Education, Wuxi 214122, China; elainewang2022@163.com (Y.W.); caimingli@jiangnan.edu.cn (C.L.); banxiaofeng521@163.com (X.B.); zhengbiaogu@jiangnan.edu.cn (Z.G.); hongyan@jiangnan.edu.cn (Y.H.); chenglichocolate@163.com (L.C.); 2 School of Food Science and Technology, Jiangnan University, Wuxi 214122, China; 3 Collaborative Innovation Center of Food Safety and Quality Control, Jiangnan University, Wuxi 214122, China

**Keywords:** maltotetraose-forming amylase, disulfide bond, thermostability, structural information, in silico tools

## Abstract

Maltooligosaccharides are a novel type of functional oligosaccharides with potential applications in food processing and can be produced by glycosyl hydrolases hydrolyzing starch. However, the main obstacle in industrial applications is the balance between the high temperature of the process and the stability of enzymes. In this study, based on the structural information and in silico tools (DSDBASE-MODIP, Disulfide by Design2 and FoldX), two disulfide bond mutants (A211C-S214C and S409C-Q412C) of maltotetraose-forming amylase from *Pseudomonas saccharophila* STB07 (MFA*_ps_*) were generated to improve its thermostability. The mutation A211C-S214C was closer to the catalytic center and showed significantly improved thermostability with a 2.6-fold improved half-life at 60 °C and the thermal transition mid-point increased by 1.6 °C, compared to the wild-type. However, the thermostability of mutant S409C-Q412C, whose mutation sites are closely to CBM20, did not change observably. Molecular dynamics simulations revealed that both disulfide bonds A211C-S214C and S409C-Q412C rigidified the overall structure of MFA*_ps_*, however, the impact on thermostability depends on the position and distance from the catalytic center.

## 1. Introduction

Maltooligosaccharides are usually composed of 2~10 α-D-glucopyranosyl units linked by α-1,4 glycosidic bonds [1]. They are a novel type of functional oligosaccharide that may be used in food processing and have health benefits for humans [2]. Maltooligosaccharide-forming amylases (MFAses, EC 3.2.1) are important components of glycosyl hydrolase family 13 (GH13) used in the production of maltooligosaccharides from starch [1]. However, many MFAses derived from mesophilic bacteria are unstable at high temperatures, which is a major problem in industrial applications, due to the fact that the maltooligosaccharides’ production involves the saccharification process at around 50~60 °C [1,2]. Higher thermostability will make this enzyme more competitive and desirable in the industry [3,4].

In proteins, disulfide bonds are formed by the oxidation of two nonadjacent cysteines, thus linking the side chains of two cysteines and their respective main peptide chains, which are vital in restricting the motion of protein unfolding or maintaining the conformations of globular structures [5]. Disulfide bond engineering is a promising and common strategy for the design of proteins to improve thermostability by substituting cysteines for a pair of wild-type residues by modifying genes. In the process of disulfide bonds design, the selection of the appropriate candidate residue pairs in the protein for site-directed mutagenesis is critical. The most common in silico tools are DSDBASE-MODIP and Disulfide by Design2 [6,7]. Based on the amino acid sequence and three-dimensional structure of proteins, the two kinds of software can accurately predict the location of disulfide bonds in protein structures, combined with the distance and dihedral angle constraints. These two tools were used by Badieyan et al. to introduce a new disulfide cross-link to the highly flexible 56-amino acid subdomain of cellulase C, which led to an increase in the half-life by 5.8-fold at 65 °C [8].

Thanks to the improvements in structure solution techniques and the development of bioinformatics tools, currently 187,423 protein structures have been identified and are available in the Protein Data Bank (PDB) database, thus providing a solid foundation for homologous modeling and structure-based molecular modification. Moreover, researchers have developed many structure prediction algorithms or software for efficient homologous modeling, including AlphaFold2, SWISS-MODEL, ROBETTA, I-TASSER and so on [9,10,11,12]. Therefore, in the absence of crystal structure information of the target protein, accurate protein structure can also be obtained by homologous modeling to predict or analyze the potential disulfide bonds in proteins.

In a fluctuating cellular environment, disulfide bonds mainly stabilize protein structure by reducing the conformational entropy of the denatured state [13]. Computation-aided stability prediction is an appealing way to speed up the disulfide bond engineering. It is possible to predict changes in the free energy of unfolding (ΔΔG = ΔG_mutant_ − ΔG_wild-type_, ΔΔG < 0 stabilizing, ΔΔG > 0 destabilizing), following single or multiple point mutation by developing different convenient tools, such as FoldX, I-Mutant, PoPMuSiC and Rosetta, [11,14,15,16,17]. Based on systematically evaluating several stability predictors, Khan et al. found that I-Mutant, D-Mutant, and FoldX provided nearly identical accuracies (60%) [18]. Wang et al. used FoldX to analyze the structure of r27RCL from *Rhizopus chinensis* and generated a quadruple mutation (S142A/D217V/Q239F/S250Y) by site-directed mutagenesis that exhibited a 41.7-fold improvement in the half-life at 60 °C [19].

However, there are still various studies which have shown that some mutated disulfide bonds contribute little to the thermal stability of proteins; it could even be that the two cysteines fail to form disulfide bonds, or the mutated residue destroys the surrounding stable structure by removing the native hydrophobic bonding, hydrogen bonding, salt bridge and other interactions within or between molecules [5,20,21]. Therefore, in addition to computer bioinformatics prediction, the location of disulfide bonds and their influence on interactions within or between molecules should also be fully considered.

In this study, we sought to enhance the thermostability of MFAses from *Pseudomonas saccharophila* STB07 (MFA*_ps_*, EC 3.2.1.60), a mesophilic enzyme, through disulfide bonds engineering. Based on the structural mode of MFA*_ps_*, we obtained a set of potential variants of MFA*_ps_* by disulfide bonds design tools DSDBASE-MODIP and Disulfide by Design2. Then, the structure-based computational design software FoldX and homologous modeling software AlphaFold2 were applied to screen thermostable variants from these predicted candidates Two mutants, A211C-S214C and S409C-Q412C, were constructed and assessed. Then, we elucidated the mechanism of the thermostability of the mutants via molecular dynamics (MD) simulation.

## 2. Materials and Methods

### 2.1. Materials

*Escherichia coli* JM109 and *Bacillus subtilis* WB600 strains were used for gene cloning and expression, respectively. The recombinant plasmid pST bearing the MFA*_ps_* gene (GenBank: X16732.1) from *Pseudomonas saccharophila* STB07 was constructed in this study [22]. Polymerase chain reaction (PCR) primers were synthesized by Genewiz (Suzhou, China). PrimeSTAR HS DNA polymerase and ClonExpress II One Step Cloning Kits were purchased from Vazyme Biotech Company, Ltd. (Nanjing, China). DNA sequencing was performed by Genewiz (Suzhou, China). His Trap HP affinity columns were purchased from GE Healthcare Life Sciences (Beijing, China). Bio-Rad protein assay reagent kits were purchased from Beyotime Biotechnology (Shanghai, China). All other materials were purchased from Sigma-Aldrich (St. Louis, MO, USA), Takara (Beijing, China), or Sinopharm Chemical Reagent (Shanghai, China).

### 2.2. Construction of Site-Directed Mutants

The site-directed mutagenesis was performed using PCR with two sets of forward and reverse primers, as described by Xie et al. in [23]. The wild-type recombinant plasmid mfa_ps_/pST was used as the PCR template. In the PCR, the primers R1 and F2 were used in the first reaction, while R2 and F1 were used in the second reaction, and intermediates L1 and L2 were produced, respectively (Table 1). The amplified products L1 and L2 were detected by 0.1% (*w*/*v*) agarose gel electrophoresis.

Next, a ClonExpress II One Step Cloning Kit was used to link the completely complementary ends of the L1 and L2. Afterwards, a chemical transformation was conducted into *E. coli* JM109 with the recombinant plasmids for manipulation and amplification. By sequencing the plasmids for mutations (Genewiz, Suzhou, China), we were able to confirm the correct mutations. Finally, the plasmids were transformed into *B. subtilis* WB600 for expression.

### 2.3. Expression and Purification of Wild-Type MFAps and Its Mutants

Wild-type MFA*_ps_* and their mutants were generated by *B. subtilis* WB600 carrying plasmid mfa_ps_/pST or variants, according to Pan et al. in [24]. Wild-type MFA*_ps_* and mutants were purified with a 5-mL HisTrap HP column (5 mL, GE Healthcare) pre-equilibrated with buffer A (10 mM Tris-HCl, 500 mM NaCl, pH 7.5). After loading, the MFA*_ps_* were eluted with buffer B (10 mM Tris-HCl, 500 mM NaCl, 300 mM imidazole, pH 7.5) at 1 mL/min. The eluent was collected and analyzed using SDS-PAGE, and the molecular weight was determined. The purified protein was dialyzed for 24 h by dialysis buffer (10 mmol/L Tris-HCl, pH 7.5) to remove saline ions and assayed for enzymatic activity and concentration [23,24]. The protein concentration was measured using the Bradford method with a Bio-Rad protein assay reagent kit [25]. Purified enzymes were stored at −80 °C.

### 2.4. Measurement of Activity and Kinetics

MFA*_ps_* activity was determined by measuring the amount of reducing sugars released from hydrolysis starch, according to Xie et al. in [26]. As the enzyme reaction mixture, 100 μL of appropriately diluted MFA*_ps_* was added to 900 μL of 10 mM NaH_2_PO_4_-Na_2_HPO_4_ buffer (pH 7.5) containing 1% (*w*/*v*) soluble starch. Reactions were incubated at 50 °C for 15 min, and then the amount of released reducing sugar was determined by the 3,5-dinitrosalicylic (DNS) acid method of Miller [27]. One unit (U) of hydrolysis activity was defined as the amount of enzyme that produced 1 μmol of reducing sugar (in terms of glucose) per minute at 50 °C.

Kinetic parameters were measured in 10 mM NaH_2_PO_4_-Na_2_HPO_4_ buffer (pH 7.5) with maltodextrin (DE = 4) concentrations ranging from 0.1 to 10 mg/mL. The catalytic activity of the enzyme was assayed under the same conditions as mentioned above.

### 2.5. Temperature Inactivation to Wild-Type MFAps and Its Mutants

Purified wild-type MFA*_ps_* and their mutants were diluted to 0.1 mg/mL with dialysis buffer. The half-life of enzymes was measured by incubating 100 μL enzymes at 50 or 60 °C. Samples were taken at different times. Subsequently, enzymes were cooled to 0 °C and the residual activity was measured at 50 °C or 60 °C.

### 2.6. Measurement of Thermal Transition Mid-Point (T_m_)

The *T_m_* of wild-type MFA*_ps_* and mutants were measured using nano-differential scanning calorimetry (DSC, TA Instruments, Inc., New Castle, Delaware, USA). The pure enzymes were dispersed in dialysis buffer and degassed before scanning. The dialysis buffer was used as a reference, and the sample volume was 0.3 mL. The instrument was balanced for 10 min before each scanning, and the pressure in the pool was maintained for 3 atm during the scanning process. The software of Launch Nano Analyze and TA Universal Analysis were used to calculate and analyze the scanning temperature. All values were determined in triplicate.

### 2.7. Homology Modeling

The homology models of wild-type and mutants were constructed according to the crystal structure of MFA*_ps_* (PDB ID: 6IWK) using the AlphaFold2 protein modeling server (https://github.com/deepmind/alphafold accessed on 1 October 2021). The structures were visualized and analyzed using the PyMOL molecular graphics system (http://www.pymol.org accessed on 1 September 2020).

### 2.8. Location of the Disulfide Bridge Sites

DSDBASE-MODIP (http://caps.ncbs.res.in/iws/modip.html accessed on 10 December 2020) and Disulfide by Design2 (http://cptweb.cpt.wayne.edu/DbD2/index.php, http://caps.ncbs.res.in/iws/modip.html accessed on 10 December 2020) were used to predict potential disulfide bridges in MFA*_ps_*.

### 2.9. ΔΔG Calculations and Mutational Scanning

The thermal stability changes of mutations compared with wild-type MFA*_ps_* were evaluated using the energy calculation function of protein design software FoldX (http://foldxsuite.crg.eu/ accessed on 7 June 2021) [28]. In this study, we performed five separate tests and compared the mean values of the ΔΔG. In general, the prediction error of FoldX is about 1 kcal/mol. The ΔΔG was used to evaluate the thermal stability of the variants and screened mutants.

### 2.10. Molecular Dynamics Simulations

A molecular dynamics simulation using Amber 16 was used to observe the structural flexibility of wild-type MFA*_ps_* and variants. The FF99SBildn11 force field was used for protein residues. Enough Na^+^ was added to neutralize the negative charges in the system. The whole system was minimized for 2000 steps, and gradually heated to 300/320/340 K and then equilibrated for 10 ps under constant T and P. Finally, a 200 ns MD simulation of proteins was conducted under constant volume and temperature (300/320/340 K). Analyses of the MD trajectories were conducted using visual molecular dynamics (VMD) and its plugins. Root mean square deviations (RMSD) and root mean square fluctuation (RMSF) were calculated for the protein backbone atoms using least-square fitting derived from the MD trajectories [29].

### 2.11. Product Analysis Using Corn Starch

Maltooligosaccharides were obtained by incubating wild-type MFA*_ps_* or mutants (20 U/g of starch) with 20% (*w*/*w*) maltodextrin (DE = 7~9) solution at 50 °C in a water-bath shaker (200 r/min) for 24 h. The reaction mixtures were boiled for 30 min to terminate the reaction, according to Pan et al. in [24]. The products were analyzed using HPAEC-PAD by comparison of pure high-performance liquid chromatography-grade glucose (G1) and maltose (G2) to maltoheptaose (G7) as standard.

### 2.12. Statistical Analysis

Experimental data reported represents the mean of triplicate measurements. The means and their standard deviations were determined by using the statistical software package from SPSS, Inc., USA. Differences resulting in values of *p* < 0.05 were considered statistically significant.

## 3. Results and Discussion

### 3.1. Structural Modeling

MFA*_ps_* from *Pseudomonas saccharophila* STB07 is a typically modular protein consisting of three regions: N-terminal catalytic domain, C-terminal carbohydrate-binding module20 (CBM20) and the linking region between the catalytic domain and CBM20, respectively. Partial crystal structures of MFA*_ps_* have been known. Zhang et al. [22] analyzed the crystal structure of MFA*_ps_* (PDB ID: 6IWK) at 1.5 Å by X-ray diffraction, which shows a 418-amino acid protein folded into a catalytic module. Unfortunately, it is thought that the CBM20 (L433~F530) at the C-terminal of MFA*_ps_* and the linker (G419~G432) linking CBM20 to the catalytic module are too flexible to obtain crystal structure. D68~G70 of loop5 (P57~S91) also has no electron density during diffraction due to its high flexibility.

**Figure 1:**
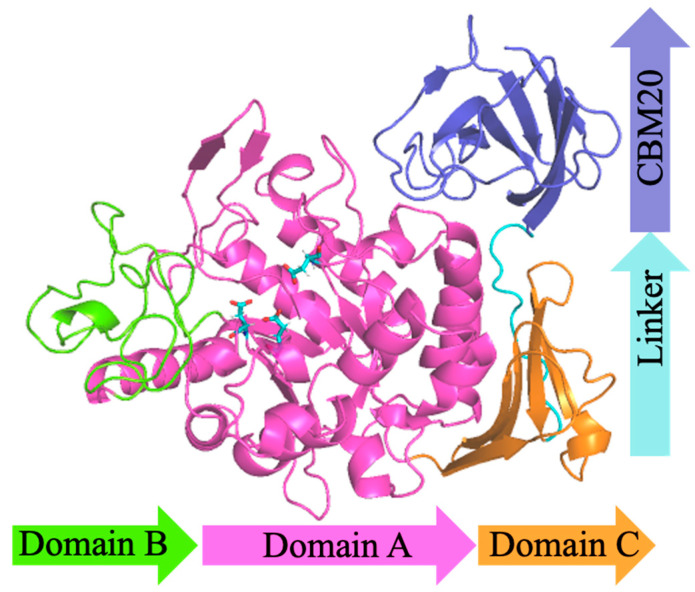
Overall structural model of MFA*_ps_*. Domains A, B, C, linker and CBM20 are rendered in blue, pink, green, cyan and blue, respectively. Catalytic residues and Ca^2+^ are represented by cyan sticks and green spheres, respectively.

**Figure 2:**
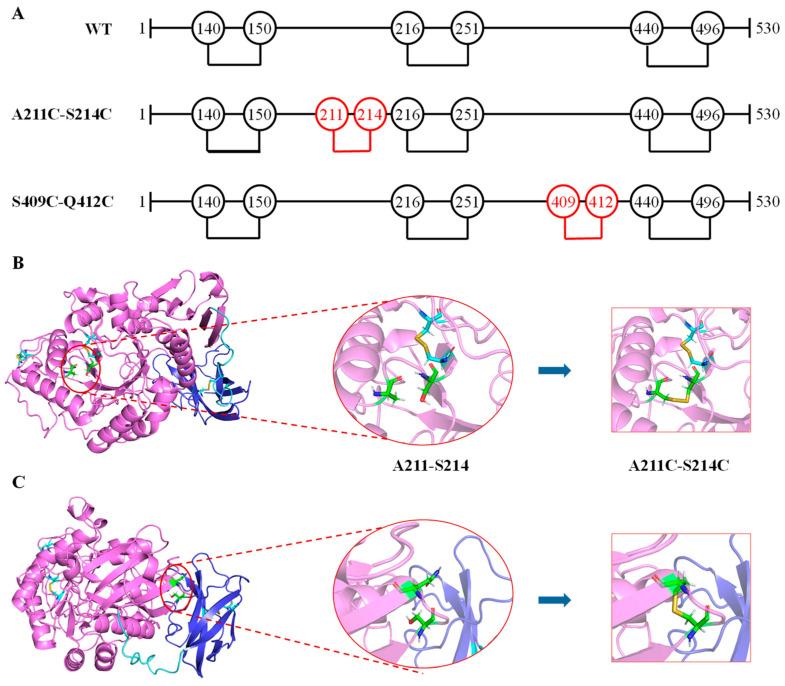
Disulfide bonds in MFA*_ps_*. (**A**) Diagram of MFA*_ps_* amino acid sequence indicating native cysteine residues (black) and new mutated residues (red) forming disulfide bonds. (**B**) Structure of variant A211C-S214C. (**C**) Structure of variant S409C-Q412C. Native and new cysteines residues are represented by cyan and green sticks, respectively.

Homology modeling is the most effective method to predict unresolved protein structures [30]. Here, we used AlphaFold2 protein modeling software to get the overall structure of MFA*_ps_* to predict possible positions for introducing disulfide bonds, as shown in Figure 1 The catalytic module consists of three domains, domains A, B, and C, respectively. Domain A (D1~N113 and H167~D360) includes a cavity containing the active-site residues (D193, E219 and D294) and is a typical (β/α)_8_-barrel structure, which is composed of alternating connections between eight α-helices (α1~α8) and eight β-strands (β1~β8) and is common in α-amylases of the GH13 family [31]. It is thought that the (β/α)_8_-barrel structure of amylases is the most widespread and stable fold in characterized and resolved enzymes [32]. Having intrinsic stability, this (β/α)_8_-barrel structure can be used as a core scaffold for molecular modifications targeting thermal stability and catalytic activity [33]. Some highly flexible regions in the domain A, such as W63~G73, S225~W232 and T295~H306, could not be used for mutagenesis because of their proximity to the active site and is probably in charge of the recognition of substrates [21]. Domain B (V114~G166), which is an extension positioned between helix α3 and strand β3, forms the substrate-binding pocket with domain A. Significantly different from the MFAse with better thermal stability, such as BSA G6-amylase from *Bacillus* sp. 707 and BstMFAse from *B. stearothermophilus* STB04, their domain B consists of five β-strands, whereas the domain B of MFA*_ps_* consists mainly of the random coil [34,35]. The domain C (S361~S418) of MFA*_ps_* is a typical Greek key motif and consists of five antiparallel β-strands. At the same time, a wild-type MFA*_ps_* contains two native disulfide bridges (140C-150 C and 216C-251 C, Figure 2A).

### 3.2. Computational Design and Screening of Disulfide Bonds

To increase the thermostability of MFA*_ps_*, the introduction of extra disulfide bridges by mutating two amino acid residues into cysteine was proposed. Based on the overall three-dimensional structure of the MFA*_ps_*, the DSDBASE-MODIP and Disulfide by Design2 online servers were used to predict potential disulfide bonds. Firstly, 132 pairs of potential disulfide binds meeting the geometric parameters in MFA*_ps_* were predicted by considering the bond lengths and bond angles of disulfide bonds. Based on the observation of the previous discussion, we realized that the C-terminal was relatively loose and the N-terminal was closely related to the binding of Ca^2+^, so the C-terminal and N-terminal amino acid residues were not included in the scope of mutation. Because too many residue pairs were still present, additional selection criteria were necessary. The protein design software FoldX uses linear combinations of energy with different weights to calculate the ΔΔG and can predict the effects of multiple point mutations on protein stability [14]. The results showed that among all potential disulfide bond mutants, A211C-S214C and S409C-Q412C not only had smaller ΔΔG but were also located in the loops that were on the protein surface and far from the catalytic center (Figure 2), thus reducing the risk of compromising the activity. The structures of A211C-S214C and S409C-Q412C were also constructed by AlphaFold2 and possible to visually form disulfide bonds, as shown in Figure 2.

### 3.3. Expression, Purification and Biochemical Characterization of MFA_ps_ and Its Mutants

**Figure 3:**
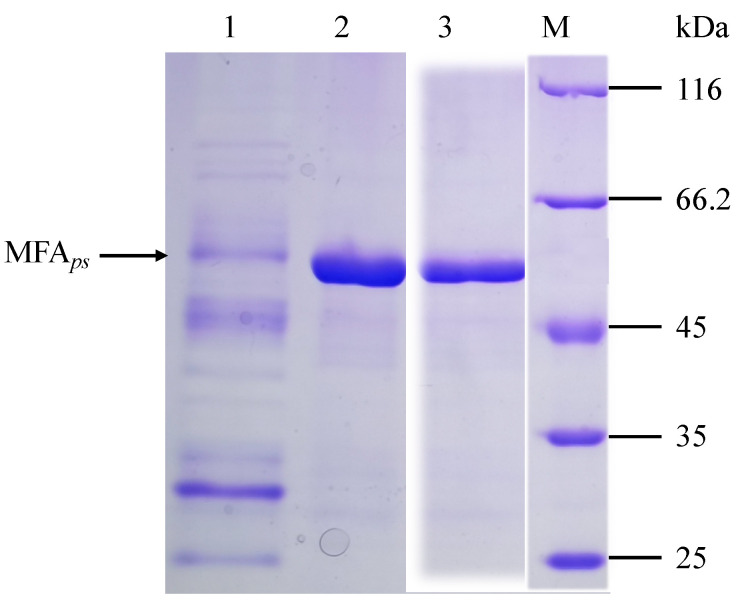
The SDS-PAGEs of culture fluids containing the recombinant forms MFA*_ps_* and purified enzymes. Lane M is marker (kDa); lane 1 is wild-type recombinant strain; lane 2 is the purified variant A211C-S214C; lane 3 is the purified variant S409C-Q412C. Black arrows indicate the band of 53 kDa.

The wild-type MFA*_ps_* and their mutants were constructed by site-directed mutagenesis, expressed in *B. subtilis* WB600 and purified for a characterization using His Trap HP affinity columns. Here, two approximately 53 kDa bands of the purified mutant A211C-S214C and S409C-Q412C (lane 2 and lane 3) in SDS-PAGE (Figure 3) were visible and in line with the theoretical molecular weight. This result demonstrated that purification was not affected by mutagenesis.

Since the factors related to protein inactivation mechanism are not considered in the calculation of ΔΔG, the effect of mutation on enzyme activity is difficult to predict and can only be verified experimentally. The catalytic activities toward starch were determined for the wild-type and mutants and the activity of the wild-type MFA*_ps_* was set as 100%. As shown in Table 2, for both mutants, activities of culture fluids displayed a 24~32% reduction. Although the specific activities of mutant S409C-Q412C were similar to wild-type MFA*_ps_* (97.1%), A211C-S214C was 18% lower than wild-type MFA*_ps_*. The reduction in the activity of mutants is probably due to the reduced expression levels. In some research, the increase in the number of disulfide bonds in proteins caused difficulties in protein folding and secretion in microorganisms, which affects enzyme activity [18].

The kinetic properties of wild-type MFA*_ps_* and mutants were determined using maltodextrin (DE = 4) as substrates. According to the Lineweaver-Burke analysis, the kinetics of wild-type and variants hydrolysis were consistent with the Michaelis-Menten equation (*R*^2^ > 0.98). The *K*_m_ and *k*_cat_ of wild-type MFA*_ps_* and variants showed no substantial differences, suggesting that these mutations did not significantly affect the interactions of the substrate with MFA*_ps_* (Table 2).

### 3.4. Effect of Mutations on Thermostability

**Figure 4:**
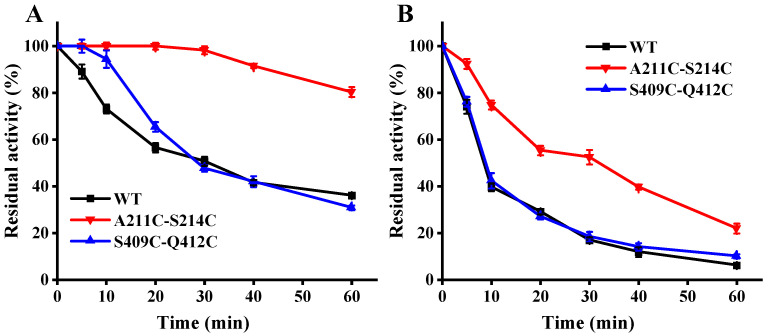
The thermo inactivation of WT (black line), A211C-S214C (red line) and S409C-Q412C (blue line) at pH 7.0. (**A**) 50 °C. (**B**) 60 °C. The activity of the enzyme before incubation was set as 100%. WT: wild-type MFA*_ps_*.

We evaluated the thermostability of the MFA*_ps_* variants from their residual activity after temporarily heating at an elevated temperature. As shown in Figure 4A, both mutants showed increased residual activity after incubation at 50 °C for 20 min, whereas mutant S409C-Q412C retained a similar activity to wild-type MFA*_ps_* after heating for 20 min. When heated at 50 °C for 1 h, the wild-type retained 36.1% activity and mutant S409C-Q412C retained a similar level of activity, only retaining 30.8% of the initial activity. In contrast, A211C-S214C showed a high residual activity of 80.4%, nearly 2.3-fold higher than that of wild-type MFA*_ps_* after heating at 50 °C. The wild-type lost activity rapidly at a higher temperature of 60 °C and maintained only 10.6% of the initial activity after 1 h (Figure 4B), and again variant A211C-S214C displayed a higher level of activity of 28.2%, a nearly 2.7-fold improvement in residual activity compared with wild-type. Mutant S409C-Q412C retained a similar activity to wild-type MFA*_ps_* after heating from beginning to end, suggesting that 60 °C was leading to more extensive thermal unfolding-induced aggregation of S409C-Q412C. The half-life for the loss of enzyme activity at 60 °C was also analyzed. As seen in Table 3, A211C-S214C had the longest half-life of 28.2 min, a 2.6-fold improvement over that of the wild-type MFA*_ps_*.

The *T_m_* was also measured for the MFA*_ps_*. The *T_m_* values for the variant S409C-Q412C were essentially unchanged compared with wild-type, whereas A211C-S214C increased the *T_m_* over that of wild-type MFA*_ps_* by 1.6 °C (Table 3). The increased *T_m_* values of the A211C-S214C were consistent with the improved residual activities observed after heating at 60 °C.

### 3.5. Analysis of Structural Stability

**Figure 5:**
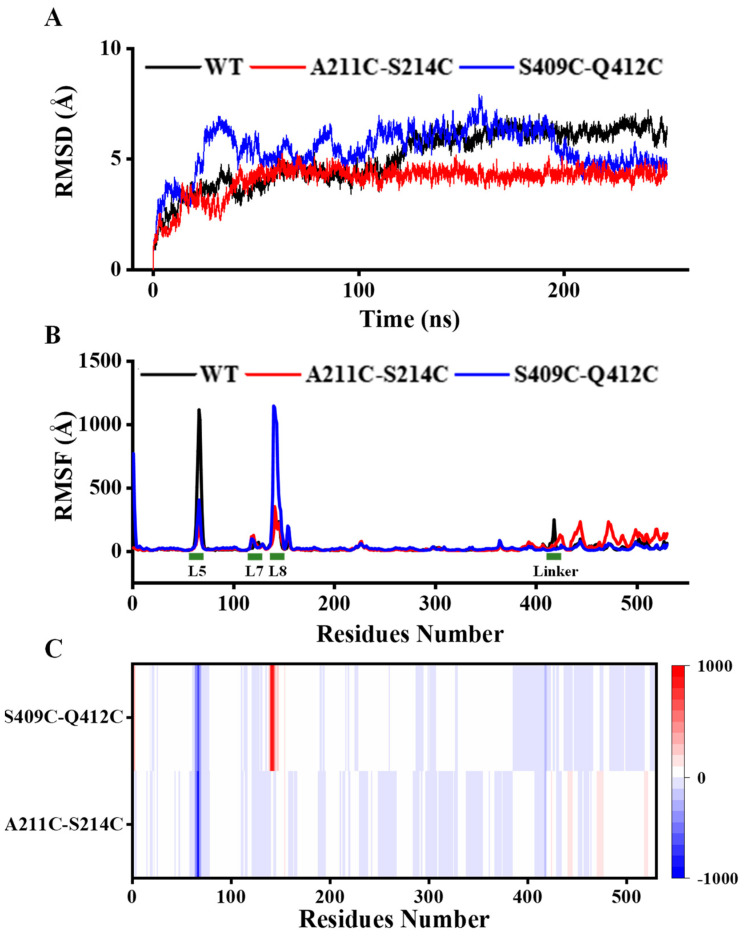
MD simulation analysis of wild-type MFA*_ps_* and mutants. (**A**) Calculation and distribution of the RMSD values. (**B**) RMSF values of each amino acid after MD simulation processes for 200 ns. MD, molecular dynamics; RMSD, root mean square deviation; RMSF, root mean square fluctuation. (**C**) Heat map showing the ΔRMSF of mutants relative to WT, highlighted red for ΔRMSF > 0, blue for ΔRMSF < 0, and white for ΔRMSF = 0, and the darker the color, the greater the absolute value of ΔRMSF. WT: wild-type MFA*_ps_*.

Combining structural difference analysis and MD simulation at 320 K, we explored the variation in flexibility of mutants. The RMSD values that were calculated for the backbones of all residues were used to explore the overall flexibility of the whole structure. A lower RMSD typically indicates a less flexible and potentially more thermostability of the overall structure [36]. As shown in Figure 5A, the RMSD values of wild-type and mutants increased significantly at the beginning of the simulation. At 50 ns, the RMSD values of mutant A211C-S214C were approximately stable at 4.5 Å until the end of the simulation. Although the RMSD values of mutant S409C-Q412C fluctuated significantly in the first 200 ns of the simulation, they gradually stabilized in 200–250 ns. At the end of the simulation, mutant S409C-Q412C also showed a lower RMSD (4.7 Å) than that of the wild-type (6.0 Å). It indicated that the introduction of an additional disulfide bond increased the rigidity of both mutants and stabilized the overall structure.

We also investigated the RMSF values for all structures at 320 K (Figure 5B) and calculated the ΔRMSF between the variants and wild-type MFA*_ps_* (Figure 5C) to understand which structural features were stabilized or destabilized in the variants and wild-type MFA*_ps_*. The white zones in heat maps (Figure 5C) indicated that the flexibility in most of the structure of variants was not changed significantly, and highlighted red or blue represents an increase or decrease in the flexibility of a local region relative to the wild-type [36].

Compared with the structural module of wild-type MFA*_ps_*, the flexibility of loop5 (P57~S91) in both mutants was significantly reduced (Figure 5B,C), especially W66~G73, which seemed to improve the stability of the overall structure by increasing the local rigidity of the loop. From the structural differences between the mutants and the wild-type MFA*_ps_*, the increase in rigidity of loop5 might be because of the combined effects caused by changes in various interaction forces. For example, there are a pair of hydrophobic interactions (F79-I157) and a pair of cationic-π interactions (F63-R61) formed in loop5 (P57~S91) of both mutants but not in wild-type MFA*_ps_*, which may make loop5 more stable.

It was worth noting that the loop8 (C140~H167) of mutant S409C-Q412C showed decreased rigidity and increased fluctuation compared to those of wild-type MFA*_ps_* (Figure 5B,C), which means that the loop8 (locally unstable) might begin local unfolding at high temperatures before the more stable overall protein structure (global stability). This is probably because the disulfide bond S409C-Q412C is distant from loop8 and only selectively stabilized the CBM20 region of the mutant structure, especially loop30, where the mutation occurred. These could explain why mutant A211C-S214C showed better thermostability than S409C-Q412C, although the same number of disulfide bonds was introduced. MD simulation revealed that the impact on the thermostability of introducing a disulfide bond is closely related to the location and the distance from the catalytic center of the new disulfide bond.

Interestingly, we also observed that mutations not only influence the dynamics of their local environment but also, in some cases, the dynamics of distant regions in the structure. Compared with the wild-type, the flexibility of loop8 (C140~H167) in S409C-Q412C was increased, while the flexibility of linker and CBM20 was decreased (Figure 5B,C). Correspondingly, we identified three hydrogen bonds (A140-A138, R137-A146, A148-A141) existing in loop8 (C140~H167) of wild-type MFA*_ps_* but not in the mutant, and a new disulfide bond S409C-Q412C in the mutant. On the other hand, the introduction of disulfide bond A211C-S214C decreased the flexibility of loop8 (C140~H167) to below that in wild-type MFA*_ps_*, while increasing the overall flexibility of the linker and CBM20 (Figure 5B,C). Combined with local structural analysis, salt bridges and hydrogen bonds greatly influenced the flexibilities of loop8 and CBM20. In the loop8 (C140~H167) of mutant A211C-S214C, there is a new hydrogen bond (A202-A152) and a new salt bridge (D151-R202), which are absent in wild-type MFA*_ps_*. On the contrary, the interaction between amino acids in CBM20 of mutant A211C-S214C is significantly reduced relative to wild-type MFA*_ps_*. Specifically, ten hydrogen bonds (G423-G421, G425-G423, N426-G424, D427-A425, G428-N426, G429-D427, E430-G428, L433-G431, V434-G432, R508-L506) and three salt bridges (H306-D503, D312-R499, R346-E430) were lost in the mutant A211C-S214C. These indicated that the influence of interaction forces in the CBM20 on the thermostability of MFA*_ps_* is less than that in the active center region.

MD simulation suggested that increasing the rigidity of the active center can more significantly improve the kinetic thermostability of the entire enzyme, as a high-energy and unstable active region (local flexibility and instability) with numerous unfavorable contacts within a more stable overall protein structure (global stability) in catalyzing reactions [30,37]. Similarly, active-site mutants showed decreased activity with a contingent increase in stability relative to wild-type β-lactamase and reduced flexibility. Moreover, Xie et al. and Zhang et al. proposed an active center stabilization strategy to improve the kinetic thermostability of *Candida antarctica* lipase B and *Candida rugosa* lipase1, respectively [38,39]. Further MD simulation confirmed that the enhanced rigidity decreased the fluctuation of the active-site residues at high temperatures [38].

### 3.6. Effect of Mutations on Maltodextrin Hydrolysis

**Figure 6:**
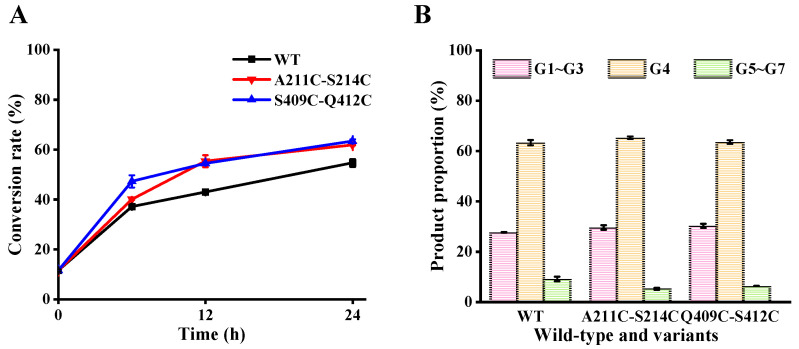
Hydrolysis product analysis of maltodextrin (DE = 7~9) by wild-type and mutants at 50 °C. (**A**) Conversion rate. (**B**) Product proportion of wild-type MFA*_ps_* or mutant hydrolyzed maltodextrin (DE = 7~9) for 24 h. The product proportion represents the mass ratio of the individual oligosaccharide to the total mass of G1 through G7. The conversion rate represents the ratio of the total mass of G1 through G7 to the original dry mass of the substrate. WT: wild-type MFA*_ps_*. G1~G3, glucose, maltose and maltotriose; G4, maltotetraose; G5~G7, maltopentaose, maltohexaose and maltoheptaose.

Product specificity is a key evaluation index for the industrial application of MFAese [26]. To explore whether the introduction of disulfide bonds changes the product specificity of MFA*_ps_*, the hydrolysis products obtained by treating maltodextrin (DE = 7~9) with wild-type MFA*_ps_* and mutant were analyzed using HPAEC-PAD (Figure 6). As shown in Figure 6A, at the initial reaction (6 h), the substrate conversion rate of S409C-Q412C was the highest, reaching 47.3%, which was about 10.1% higher than that of wild-type. After hydrolysis for 24 h, the conversion rate of the wild-type was 54.67%, while the conversion rates of A211C-S214C and S409C-Q412C increased to 61.8% and 63.4%, respectively. Maybe it is because four mutant sites are far away from the active center, which has no significant effect on the binding of substrate and enzyme center. Moreover, the additional disulfide bond leads to an increase in the thermostability of the mutants, which prolongs the time for the mutants to efficiently hydrolyzed the substrate, thus increasing the conversion rate.

**Figure 7:**
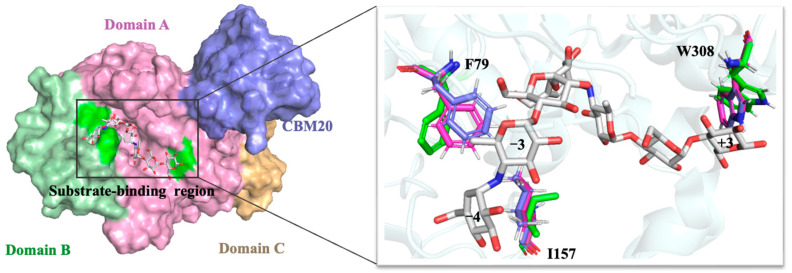
Structural changes of MFA*_ps_* near nonreducing end of the substrate. Residues of wild-type MFA*_ps_*., A211C-S214C and S409C-Q412C are represented by green, pink and purple sticks, respectively. The pG7 molecules are shown in stick representation with carbon atoms in gray.

After wild-type MFA*_ps_* hydrolyzed maltodextrin for 24 h, the predominant product was maltotetraose (G4, 63.31%). Mutants A211C-S214C (G4, 63.31%) and S409C-Q412C exhibited a product distribution like that of the wild-type, producing G1~G7. Zhang et al. revealed that the degradation of the substrate by MFA*_ps_* follows an exo-type action pattern, which means that MFA*_ps_* hydrolyze maltodextrin from the nonreducing end and usually show excellent specificity for a single product, even during the initial stage of the reaction [22]. In addition, there are four glycone-binding subsites (−4 to −1) on the nonreducing side, and the enzyme-substrate interactions at subsite −4 are essential to the production of G4. That is why the maltodextrin was primarily hydrolyzed to G4 by MFA*_ps_*. In the homology models (Figure 7), we found that the side chain of Phe79 of the A211C-S214C and S409C-Q412C mutants formed a new hydrophobic interaction with Ile157, resulting in a hydrophobic accumulation between Phe79 and glucose residue of pG7 at subsite −3 compared to wild-type, which may be beneficial to the location and stability of the pG7′s nonreducing end at the active cleft. The results of this study support the proposition that binding of the substrate’s nonreducing end in the nonreducing end-subsite of the MFAses active center affects its product specificity and substrate conversion rate [26].

## 4. Conclusions

MFA*_ps_* may has great potential application value in the industrial production of G4 due to its high product specificity. However, the main draw-back in the industrial application of MFA*_ps_* is to obtain a balance between the high temperature of process conditions and thermal stability. In this study, the thermostability of MFA*_ps_* was studied by introducing a disulfide bond that was found to play crucial roles in the folding and thermostability of proteins in previous studies. Nevertheless, the introduction of additional disulfide bonds by site-directed mutagenesis does not represent the improvement of thermostability, due to the risk of compromising the intrinsic activity and stability caused by the disulfide bonds at an inappropriate location. Compared to disulfide bond S409C-Q412C, A211C-S214C, which was closer to the catalytic center, showed a better effect on thermostability. The half-life at 60 °C of mutant A211C-S214C was 2.6-fold higher than that of the wild-type. Mutant A211C-S214C, with improved thermal stability, is probably better able to meet the needs of industrial production than the wild-type. The additional introduction of disulfide bonds not only affected the overall protein fluctuation but also selectively improved the protein local stability, as demonstrated by the RMSF analysis of wild-type and mutants. Moreover, introducing disulfide bonds to increase the rigidity of the active center can significantly improve the kinetic thermostability of MFA*_ps_*. This work provides a practical strategy for disulfide bond engineering that could be useful for improving the thermostability of other microbial enzymes.

## Figures and Tables

**Table 1:** Primers used for desired mutagenesis.

Mutants	Primer Sequence ^A^
A211C-S214C	R1 5′-tgtGACAGCtgtTTCTGCGTTGGCGAGCTGTGG-3′ R2 5′-acaGCTGTCacaGCTGTCGCTCATCCAGCTG-3′
S409C-Q412C	R1 5′-tgtAACGGCtgtGTGCGCGTCTGGCGCAGC-3′
	R1 5′-acaGCCGTTacaGGCGTTGACCGCCTCGCT-3′
All mutants ^B^	F1 5′-AAGTAGCGAAAACTCGTATCCTTCT-3′ F2 5′-AGAAGGATACGAGTTTTCGCTACTT-3′

^A^ Nucleotide sequences corresponding to the mutated amino acids were underlined and lowercase. ^B^ All mutants used the same F1 and F2.

**Table 2:** Activities and kinetic parameters for mutants compared to wild-type MFA*_ps_* on maltodextrin (DE = 4) at 50 °C.

Enzyme	Relative Activity ^A,B^ (%)	Specific Activity ^B^ (U/mg)	*K*_m_^B^ (mg/mL)	*V*_max_^B^ (μmol·min^−1^·mL^−1^*)*	*k*_cat_^B^ (×10^3^ sec^−1^)	*k*_cat_/*K*_m_^B^ (×10^3^ mL·mg^−1^·sec^−1^)
Wild-type	100 ^c^	427.5 ± 24.6 ^b^	2.48 ± 0.08 ^b^	6.90 ± 0.37 ^a^	21.90 ± 0.49 ^b^	8.83 ± 0.10 ^a^
A211C-S214C	68 ^a^	349.3 ± 12.7 ^a^	2.29 ± 0.17 ^a^	6.43 ± 0.10 ^a^	21.05 ± 0.54 ^a^	9.19 ± 0.18 ^b^
S409C-Q412C	76 ^b^	415.2 ± 14.8 ^b^	2.23 ± 0.13 ^a^	6.29 ± 0.42 ^a^	22.13 ± 0.68 ^b^	9.91 ± 0.09 ^c^

^A^ The activity of the wild-type MFA*_ps_* was set as 100%. ^B^ Each value represents the mean of three independent measurements. Data with different superscript letters within a column are significantly different (*p* < 0.05).

**Table 3:** Half-life at 60 °C and *T_m_* of wild-type MFA*_ps_* and mutants.

Enzyme	Half-live at 60 °C (min) ^A^	*T_m_* (°C) ^A^
Wild-type	10.6 ± 0.4 ^a^	57.6 ± 0.2 ^a^
A211C-S214C	28.2 ± 0.2 ^c^	59.2 ± 0.1 ^c^
S409C-Q412C	11.6 ± 0.3 ^b^	58.0 ± 0.1 ^b^

^A^ Each value represents the mean of three independent measurements. Data with different superscript letters within a column are significantly different (*p* < 0.05).

## Data Availability

The data presented in this study are available on request from the corresponding author. The data are not publicly available due to ongoing studies.
